# Intestinal Coccidian Infections in Cancer Patients: A Case Series

**DOI:** 10.7759/cureus.38256

**Published:** 2023-04-28

**Authors:** Nathan Einhorn, Isis Lamphier, Olga Klinkova, Aliyah Baluch, Yanina Pasikhova, John Greene

**Affiliations:** 1 Infectious Diseases, University of South Florida Morsani College of Medicine, Tampa, USA; 2 Infection Control, Moffitt Cancer Center, Tampa, USA; 3 Infectious Diseases, Moffitt Cancer Center, Tampa, USA; 4 Clinical Pharmacy, Moffitt Cancer Center, Tampa, USA; 5 Internal Medicine, Moffitt Cancer Center, Tampa, USA

**Keywords:** infectious disease medicine, coccidia, microsporidia, cystoisospora belli, cyclospora, cryptosporidium infection

## Abstract

Introduction

Coccidian protozoa and microsporidian fungi are opportunistic pathogens increasingly implicated in infections in immunosuppressed individuals. These parasites typically infect the intestinal epithelium, resulting in secretory diarrhea and malabsorption. The disease burden and timeline are both greater and longer among immunosuppressed patients. Therapeutic options for immunocompromised individuals are limited. As a result, we wanted to better characterize the disease course and treatment efficacy of these parasitic gastrointestinal infections.

Methods

We performed a single-center, retrospective MedMined (BD Healthsight Analytics, Birmingham, AL, USA) chart review of patients between January 2012 and June 2022 diagnosed with coccidian or microsporidian infections. Relevant data were collected from Cerner’s PowerChart (Oracle Cerner, Austin, TX, USA). Descriptive analysis was performed with IBM SPSS Statistics (IBM Corp., Armonk, NY, USA), and Microsoft Excel (Microsoft, Redmond, WA, USA) was used to generate graphs and tables.

Results

In these 10 years, there were 17 patients with *Cryptosporidium* infections, four with *Cyclospora* infections, and no positive cultures for *Cystoisospora belli* or microsporidian infections. In both infections, the majority of patients experienced diarrhea, fatigue, and nausea, with vomiting, abdominal pain, appetite loss, weight loss, and fever occurring to a lesser degree. Nitazoxanide was the most common treatment for *Cryptosporidium*, while trimethoprim-sulfamethoxazole or ciprofloxacin were the treatments of choice for *Cyclospora*. Of the *Cryptosporidium* infections, three received combination therapy with azithromycin, immunoreconstitution, or IV immunoglobulins. Among the four *Cyclospora*-infected patients, one received combination therapy of ciprofloxacin and trimethoprim-sulfamethoxazole. Treatment lasted around two weeks, and 88% of *Cryptosporidium* patients and 75% of *Cyclospora* patients had a resolution of symptoms.

Conclusion

The most detected coccidian infection was *Cryptosporidium,* followed by *Cyclospora*, with the lack of *Cystoisospora* or microsporidian infections likely due to diagnostic limitations and prevalence. *Cryptosporidium* and *Cyclospora* likely caused their associated symptoms in most cases, with other possible etiologies, including graft-versus-host disease, medications, and feeding tubes. The small number of patients receiving combination therapy prohibited a comparison with monotherapy. In our patient population, though, there was a clinical response to treatment despite immunosuppression. While promising, additional randomized control experiments are required to fully understand the efficacy of parasitic treatments.

## Introduction

The unicellular coccidia *Cryptosporidium*, *Cyclospora*, *Cystoisospora belli*, and the spore-forming Microsporidia *Enterocytozoon bieneusi* (*E. bieneusi*) and *Encephalitozoon* spp. are parasites that typically infect intestinal epithelium, resulting in secretory diarrhea and malabsorption [1]. These coccidian protozoa and microsporidian fungi are associated with exposure to contaminated food and water [2,3]. A 2012 World Health Organization report demonstrated that *Cyclospora cayetanensis* and *Cryptosporidium* are among the top 15 global foodborne parasites [3]. With this rise in prevalence, these opportunistic pathogens are increasingly implicated in infections in immunosuppressed individuals [4,5]. A meta-analysis of 131 studies found that HIV-infected people presenting with diarrhea in low-income countries have a high prevalence of Microsporidia, *Cryptosporidium*, and *Cystoisospora* infections [5]. In a group of 62 immunosuppressed children experiencing diarrhea, *Cryptosporidium*, *Cyclospora*, and *Cystoisospora belli* were present in 22.5%, 9.6%, and 3.2% of cases, respectively [4]. While enteric infections by these organisms in immunocompetent individuals are often self-limiting, the disease burden and timeline are both greater and longer among patients with HIV, receiving cancer treatment, or undergoing organ transplantation [6,7]. For example, diarrhea duration and severity are greater in acute lymphoblastic leukemia (ALL) patients with stool-positive *Cryptosporidium* oocysts than in those with non-*Cryptosporidium* diarrhea [8]. Patients with HIV infected by *C. cayetanensis* experienced more weight loss and had a prolonged illness course relative to non-HIV patients [9]. Unfortunately, the therapeutic options for immunocompromised individuals are limited and currently not well-studied [10]. While immune reconstitution and effective antiretroviral therapy for HIV patients are generally sufficient to control these infections, there is no current effective commercial therapy for major pathogens like *E. bieneusi*, despite alternative treatments with nitazoxanide and albendazole [11]. Similarly, immunocompetent patients with coccidian infections may respond well to treatments like nitazoxanide, trimethoprim-sulfamethoxazole, or ciprofloxacin, but immunosuppressed individuals face a more uncertain path [12,13]. Furthermore, there is little data on the efficacy of combination versus monotherapy in this patient population. Thus, we performed a retrospective chart review of cancer patients treated at H. Lee Moffitt Cancer Center between 2012 and 2022 to better characterize the disease course and treatment efficacy of these parasitic gastrointestinal infections. By elucidating the different therapies, we hope to find instances of combination treatments that may provide a direction for future research.

## Materials and methods

We conducted a single-center, retrospective chart review of patients between January 2012 and June 2022 diagnosed with coccidian or microsporidian infections at the H. Lee Moffitt Cancer Center and Research Institute in Tampa, FL. University of South Florida Institutional Review Board approval was obtained before the start of the study - IRB ID STUDY004083. A MedMined (BD Healthsight Analytics, Birmingham, AL, USA) search was completed to identify patients with positive results for *Cryptosporidium*, *Cyclospora*, *Cystoisospora belli*, *E. bieneusi*, or *Encephalitozoon* spp. Tests used to identify positive results included stool-sourced enzyme immunoassays or stool-sourced gastrointestinal pathogen panels (GIP) by multiplex PCR (BioFire Diagnostics, Salt Lake City, UT, USA). Patients included in this study were 18 years or older, had a diagnosis of malignancy, and had a positive test for one or more of the above-mentioned protozoan or fungal infections. The relevant data were collected from Moffitt Cancer Center’s institutional databases of Cerner’s PowerChart (Oracle Cerner, Austin, TX, USA) and GE PACS (GE Healthcare, Chicago, IL, USA). Collected variables included age, sex, demographics, medical history, history of transplant, presenting symptoms and duration, diagnostic methods, infection diagnosis, underlying malignancy, etiology of immunocompromised state, pertinent lab values at diagnosis, treatment type and duration, clinical outcomes, coinfections, and complications. The immunocompromised state was determined by the presence of cancer treatment, steroids, other immunosuppressive medications, or infections that lower immunity, like HIV/AIDS. Clinical outcomes were determined by the resolution of symptoms since Moffitt Cancer Center does not perform tests of cure as they are not indicated. We conducted a descriptive analysis using IBM SPSS Statistics (IBM Corp., Armonk, NY, USA) to find the mean, median, and standard deviation for any numerical data. Microsoft Excel (Microsoft, Redmond, WA, USA) was used to generate graphs and tables. 

## Results

There were total of 18 *Cryptosporidium* and four *Cyclospora* infections in the 10-year study period. There were no patients with *Cystoisospora belli* or microsporidian infections, likely due to disease prevalence and diagnostic restrictions. From the 18 *Cryptosporidium* patients reviewed, 17 were true infections, and one patient had a false positive result due to contamination. One *Cryptosporidium*­-infected patient presented for reinfection several years later. Among the true-positive cases, three patients were identified by stool-sourced GIP by multiplex PCR testing, and 14 were identified by stool-sourced enzyme immunoassay. By ethnicity, eight of these patients identified as non-Hispanic, six as Hispanic, and three did not provide a response. By race, 16 of these patients described themselves as white, and one identified as African American. 76% (13/17) of *Cryptosporidium* patients were female, and 24% (4/17) were male. Patient characteristics are included in Table 1.

**Table 1:** Patient Characteristics GVHD: Graft-Versus-Host-Disease

	Cryptosporidium	Cyclospora
Male	4	3
Female	13	1
Age, mean ± SD	58±12	62±6
Race		
White	16	3
African American	1	0
American Indian	0	1
Season		
Fall	3	0
Winter	5	0
Spring	4	0
Summer	5	4
Primary Diagnosis		
Hematological malignancies	13	6
Solid Organ Tumors	8	1
Primary Amyloidosis	1	0
Malignancy Status		
Remission	4	3
Active	13	1
Immuno-Status		
Immunocompromised	15	3
Immunocompetent	2	1
History of Transplant		
Transplant	9	3
No Transplant	8	1
GVHD Status		
GVHD Present	5	0
GVHD Absent	12	4

Most (13/17) of these patients presented with at least one active malignancy. The vast majority of these patients (15/17) were immunocompromised. The most common malignancies included solid organ malignancies (8/17), acute myeloid leukemia (4/17), and multiple myeloma (2/17). A further breakdown of malignancy types is included in Table 2.

**Table 2:** Patient Malignancies AML: Acute Myeloid Leukemia; ALL: Acute Lymphoblastic Leukemia; MDS: Myelodysplastic Syndrome

Primary Diagnosis, n	Cryptosporidium	Cyclospora
Hematological malignancies	13	6
AML	4	1
ALL	1	0
MDS	1	3
Mantle Cell Lymphoma	1	1
Myelofibrosis	1	1
Myeloproliferative neoplasm	1	0
Multiple Myeloma	2	0
Hodgkin’s Lymphoma	1	0
Diffuse Large B Cell Lymphoma	1	0
Carcinoma	8	1
Cervical Cancer	1	0
Vaginal Adenocarcinoma	1	0
Squamous Cell Carcinoma	2	1
Breast Cancer	1	0
Brain Carcinoma	1	0
Iliac Bone Carcinoma	1	0
Pancreatic Adenocarcinoma	1	0
Primary Amyloidosis	1	0

**Figure 1:**
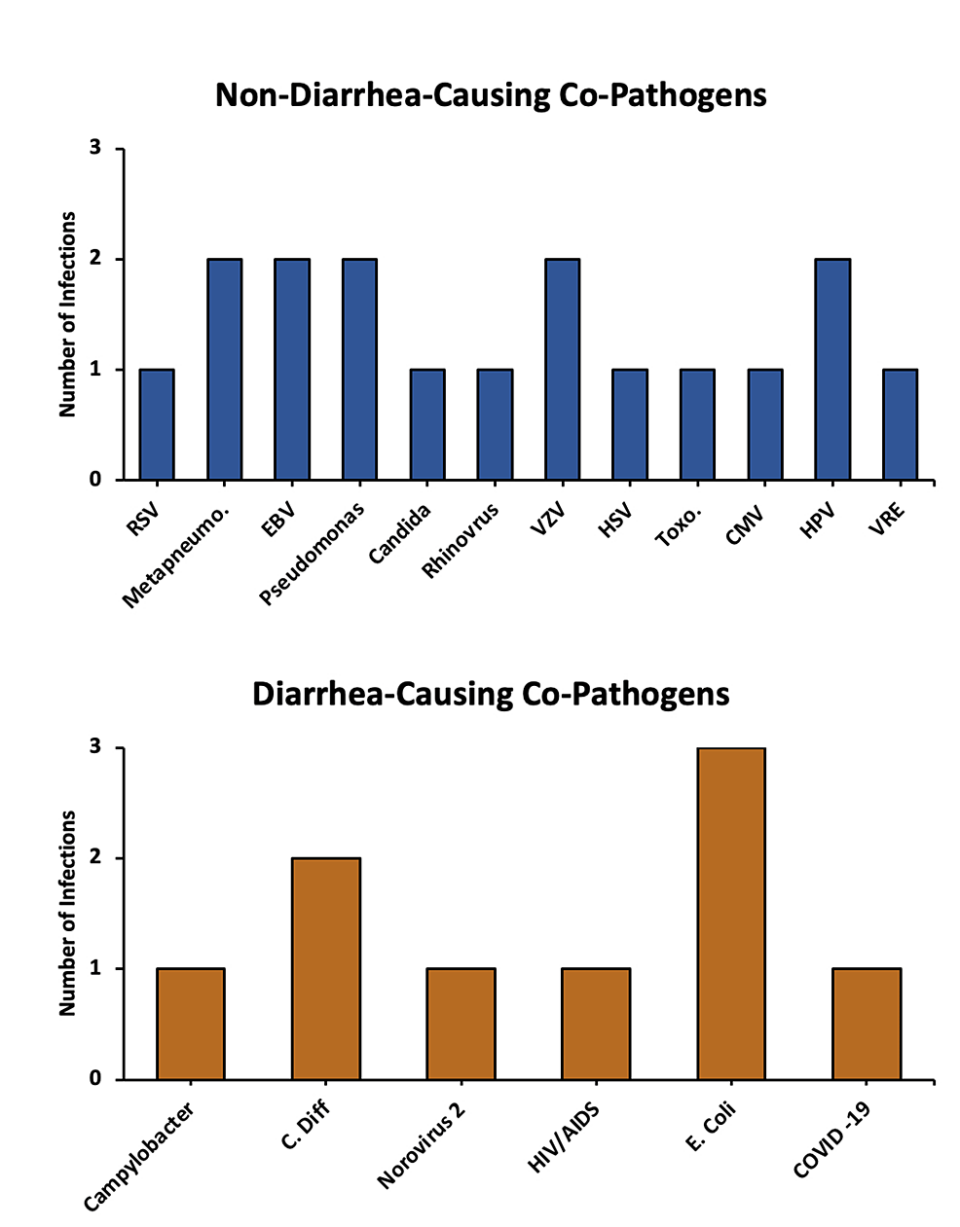
Bar charts of diarrhea-causing vs. non-diarrhea-causing co-pathogens Co-isolates found in patients infected with *Cryptosporidium* and *Cyclospora* are separated by their ability to induce diarrhea.

Over half (9/17) of the patients infected with *Cryptosporidium* had a history of allogeneic hematopoietic stem cell transplantation. Around 70% (12/17) of patients did not have any evidence of graft-versus-host disease (GVHD), while four (23%) patients presented with grade one and one patient with grade four GVHD. At the time of parasitic infection, co-isolates were identified in most patients (13/17), including pathogens like *Campylobacter jejuni* and *Clostridioides difficile*. Additional co-pathogens in both *Cryptosporidium* and *Cyclospora* cases are presented in Figure 1.

**Figure 2:**
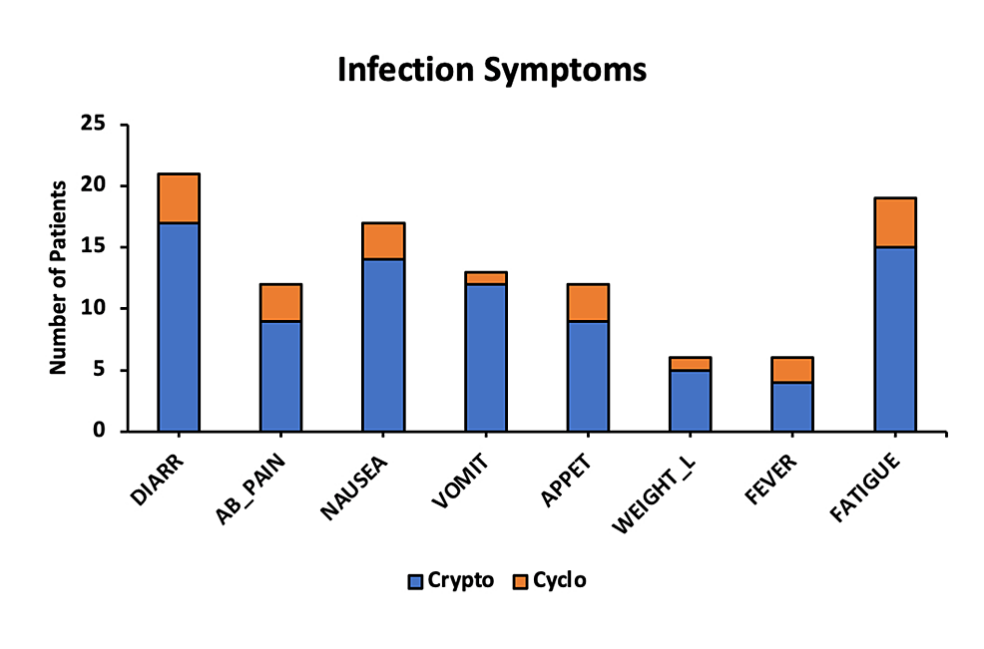
Symptoms of a Cryptosporidium or Cyclospora Infection Symptoms caused by *Cryptosporidium* and *Cyclospora* in patients treated at Moffitt Cancer Center. DIARR: diarrhea; AB_PAIN: abdominal pain; APPET: loss of appetite; WEIGHT_L: weight loss; Crypto: *Cryptosporidium*; Cyclo: *Cyclospora*.

All *Cryptosporidium* patients experienced watery diarrhea with associated symptoms of fatigue (15/17), nausea (14/17), vomiting (12/17), loss of appetite (9/17), abdominal pain (9/17), weight loss (5/17), and fever (4/17), as shown in Figure 2.

The duration of the parasitic infection and its symptoms varied greatly (average duration = 29 days, standard deviation (SD) = 27 days). There was no significant seasonality observed. In terms of primary treatment, 11 of 17 patients received monotherapy with nitazoxanide, and one of 17 received azithromycin. The combination therapies of nitazoxanide with azithromycin, immuno-reconstitution (prednisone taper), or IV immunoglobulins were each administered to one patient. Primary treatment regimens are included in Table 3.

**Table 3:** Primary Treatment Regimen TMP-SMX: trimethoprim-sulfamethoxazole; BID: twice a day; QD: once a day; n/a: not applicable; Am: antimicrobial; NAm: non-antimicrobial

Parasite	Am/ NAm	Primary Regimen	Dosage	No. Patients Monotherapy (%)	No. Patients Combination Therapy (%)	Average Duration of Therapy
Cryptosporidium	Am	Nitazoxanide	500mg BID	11 (69%)	3 (19%)	14 days
		Azithromycin	250-500mg QD	1 (6%)	1 (6%)	15 days
	NAm	IVIg	10% IVIg	0	1 (6%)	1 day
		Prednisone Taper	20mg to 10mg to 5mg QD	0	1 (6%)	48 days
Cyclospora	Am	Ciprofloxacin	500mg BID	2 (50%)	1 (25%)	14 days
		TMP-SMX	160mg/800mg QD or BID	1 (25%)	1 (25%)	12 days

Many patients (12/17) received additional treatment for symptom control, such as loperamide, IV saline rehydration, opium tincture, octreotide, dicycloverine hydrochloride, diphenoxylate/atropine, and/or ondansetron. Two patients did not receive any *Cryptosporidium*-specific treatment since one patient’s symptoms resolved following treatment of GVHD, and the other patient’s symptoms were believed to be caused by carfilzomib infusion. *Cryptosporidium* treatment lasted, on average, 13 days (SD 10.5 days), with 88% (15/17) of patients experiencing resolution of symptoms. Among patients receiving combination therapies, one patient who received nitazoxanide and azithromycin died due to stage four GVHD, while the other two patients who received either IV immunoglobulin or prednisone taper had a resolution of symptoms. Four years following the initial infection, one patient experienced reinfection of *Cryptosporidium* with similar symptoms of diarrhea and was placed on a successful three-day course of nitazoxanide. 

Among the four *Cyclospora* infections, all were identified by stool-sourced GIP PCR testing. These patients were non-Hispanic (4/4), predominantly white (3/4), male (3/4), and experienced infections during the summer months (4/4). Half (2/4) of these patients had more than one malignancy at the time of infection, and the most common malignancy in these patients was myelodysplastic syndrome (3/4). A majority (3/4) were immunocompromised. 75% (3/4) of patients infected with *Cyclospora* had a history of allogeneic hematopoietic stem cell transplant; none had GVHD. 50% (2/4) of these cases had a co-pathogen isolated, in which one patient had varicella-zoster virus present in their stool sample, and another had metapneumovirus present in their nasopharynx respiratory viral panel PCR. Like the *Cryptosporidium* cases, all *Cyclospora* patients experienced watery diarrhea with fatigue (4/4), nausea (3/4), vomiting (1/4), loss of appetite (3/4), abdominal pain (3/4), weight loss (1/4), and fever (4/4). The duration of symptoms in these four patients lasted, on average, 22 days, with a standard deviation of seven days. Trimethoprim-sulfamethoxazole and ciprofloxacin were utilized for the treatment of *Cyclospora*. Three patients received monotherapy of ciprofloxacin (2/4) or trimethoprim-sulfamethoxazole (1/4), while one patient received combination therapy of the two drugs. Patients were on these therapies for an average of 12.5 days (SD 1.7 days), and 75% (3/4) of these patients had a resolution of symptoms, while the one patient who was receiving combination therapy was lost to follow-up. 

## Discussion

In this retrospective chart review, we described the clinical characteristics, outcomes, and treatments of 21 patients with coccidian infections spanning 10 years at Moffitt Cancer Center. The most commonly detected coccidian infection was *Cryptosporidium,* followed by *Cyclospora*, with no *Cystoisospora* or microsporidian infections identified by diagnostic methods used in our institution. The lack of microsporidian and *Cystoisospora* infections in our patient population may be due to disease prevalence and/or diagnostic restrictions rather than a true absence of infection. With microsporidian infections, one analysis of 101 patients with hematological malignancies in India found that 2% of patients were infected with a microsporidian parasite, while another study of 40 adult neutropenic patients with acute leukemia in Egypt showed a microsporidian prevalence of 25% [14,15]. Since the microsporidian prevalence can be sufficiently high for detection, the issue may lie with diagnostic methods. For many years, diagnosis relied upon light microscopy with modified trichrome stain; labs must be notified to search for Microsporidia since routine examinations for ova and parasites do not detect Microsporidia. Furthermore, the few prior cases of Microsporidia detected at Moffitt before this study’s period were detected by endoscopic biopsy with histopathology examination of the tissue. *Cystoisospora* not only has a low prevalence, but the intermittent, irregular poor shedding of oocysts requires multiple stool samples for an accurate diagnosis [16]. Although most coccidia faces similar diagnostic obstacles, the prevalence and danger of *Cryptosporidium* relative to other coccidia make it a more sought-after target when evaluating for illness in susceptible individuals [17]. A study of 54 adult cancer patients receiving chemotherapy and experiencing diarrhea at King Khalid University showed a 70% prevalence rate for *Cryptosporidium *[18]. The older immunocompromised patient population of Moffitt Cancer Center requires awareness of this parasite, so if a patient is experiencing prolonged diarrhea that is thought to be community-acquired, then tests will be ordered for *Cryptosporidium*. Fortunately, multiplex PCR has shown increased efficacy in the concurrent detection of opportunistic eukaryotic infections like *Cryptosporidium*, *Cyclospora*, Microsporidia, and *Cystoisospora* [17,19]. Although Microsporidia and *Cystoisospora *are not currently included in the standard GIP multiplex PCR, their inclusion would reveal the true prevalence of these infections.

Of note, while the summer seasonality of the identified *Cyclospora* cases in our study coincides with current literature, the even seasonal distribution of *Cryptosporidium* cases does not follow the classic summer peak pattern [20,21]. However, an increase in temperature and precipitation predicts an increase in cryptosporidiosis [22]. This study was performed in Tampa, Florida, which has a humid subtropical climate throughout the year, which may explain the distribution. 

One finding that also requires further elucidation is the greater prevalence of women over men in the *Cryptosporidium *group. Explanations for this observation may include innate sex differences or accepted gender roles. A cross-sectional study of 300 urban dairy farming households in Nairobi found that *Cryptosporidium* was influenced by gender, age, and household roles since women had greater exposure to cattle feces and took care of sick people, increasing their risk of cryptosporidiosis [23]. While a direct translation of these results to other cultures and societies may be difficult, this points toward the possibility of social obligations playing a role in *Cryptosporidium* infection. However, some literature solely evaluating patients in a clinical setting often does not find a relationship between *Cryptosporidium* infection and sex [24]. Studies with larger sample sizes that explicitly consider these social factors are needed to elucidate the possible sex- or gender-related relationships in these infections. 

In this patient population, it is necessary to determine if the cardinal symptom of secretory diarrhea was caused by the parasitic infections. Based on the attending physicians’ interpretations, *Cryptosporidium* likely caused diarrhea in 59% (10/17) of cases, while *Cyclospora* caused it in 75% (3/4) of cases. The other cases were related to GVHD, chemotherapy, and a feeding tube. Although many co-isolates were discovered, as shown in Figure 1, it is not believed that these pathogens contributed to the diarrhea; rather, these co-isolates may have been colonizers, pathogens causing non-gastrointestinal symptoms, or contaminants. Since many gastrointestinal infections are transmitted via similar pathways, it is not surprising to find co-isolates, though the literature on such relationships with coccidia is limited [25]. One patient in the *Cryptosporidium* cohort had a reinfection four years later, raising the question of whether *Cryptosporidium* itself could have been a colonizer rather than a new infection. One study with mice demonstrated this possibility by colonizing the small intestines with a commensal strain of *Cryptosporidium tyzzeri *[26]. Scant evidence exists for human colonization, though. Given the length of time between infections for the patient, it seems more likely to be discrete infections, since reinfection is possible even one year after initial exposure [27].

Evaluation of therapy in this study shows an apparent clinical response to treatment despite immunosuppression in both *Cryptosporidium*- and *Cyclospora*-infected patients. Nitazoxanide was successful in resolving symptoms in many of our *Cryptosporidium* patients. Although the literature is limited, there are a few small studies showing similarly positive results for immunocompromised individuals [7,8]. In six pediatric solid organ transplant recipients, gastroenteritis from *Cryptosporidium* resolved following 14 days of treatment with nitazoxanide and rehydration [7]. Three of five *Cryptosporidium*-positive children with ALL responded to nitazoxanide, while the other two required the addition of azithromycin [8]. Unfortunately, a meta-analysis of nitazoxanide’s effectiveness on cryptosporidiosis in HIV-seropositive patients did not find a reduction in the duration or frequency of diarrhea [28]. While nitazoxanide is the first-line treatment, replacement or supplementation with azithromycin, paromomycin, rifaximin, or spiramycin may be possible [10]. A study of children with ALL on chemotherapy experiencing severe diarrhea from *Cryptosporidium* responded well to 10-day courses of paromomycin or azithromycin therapy [29]. Combination treatment of cryptosporidiosis with azithromycin and paromomycin was associated with a significant reduction in oocyst excretion and some clinical improvement in eight patients with AIDS [30]. Some of these studies of azithromycin and paromomycin as first-line treatments are from the 1990s and early 2000s, which was before the establishment of nitazoxanide as the drug of choice for *Cryptosporidium*. The patient in our study undergoing nitazoxanide and azithromycin combination therapy died from GVHD, so we were not able to determine the efficacy of this therapy. Improvements were observed in two patients when nitazoxanide was combined with either immune reconstitution or IVIg. IVIg alone has shown some effectiveness at reducing the disease burden of cryptosporidiosis, but there are not enough studies examining combination therapy with nitazoxanide [31]. In immunocompetent patients, the primary treatment for *Cyclospora* infection is trimethoprim-sulfamethoxazole (TMP-SMX). There is some efficacy of this drug with increased dosage and duration in immunosuppressed individuals. Additionally, ciprofloxacin was also found to be an acceptable means of treating cyclosporiasis in patients intolerant of sulfonamides, but to a lesser effect than TMP-SMX [32]. While there are no widely accepted practices for managing these infected immunocompromised patients, the American Society of Transplantation Infectious Diseases Community of Practices has attempted to establish treatment guidelines for these parasites in solid-organ transplant recipients, which include supportive care and the treatments previously discussed [33]. Although most literature points to TMP-SMX as first-line treatment for *Cyclospora*, 50% (2/4) of our patients received ciprofloxacin alone with resolution of symptoms. One patient had a sulfa allergy, prohibiting the use of TMP-SMX, while no clinical indication was provided for the use of ciprofloxacin alone in the other patient. We were not able to determine the therapeutic effects of combination therapy because of a lack of follow-up. Additionally, it was difficult to find studies that evaluated combination therapy of TMP-SMX with ciprofloxacin or other treatments, likely due to the more benign nature of this infection. Although data examining monotherapy and combination therapy for both *Cryptosporidium* and *Cyclospora* is sparse, determining the efficacy of combination therapy may be prudent when considering immunocompromised patients that are refractory to monotherapy.

## Conclusions

Addressing the mounting healthcare issues caused by coccidian and microsporidian infections is no simple task. The true global burden of these diseases is not known due to a lack of simple, inexpensive diagnostic tools and an underappreciation of their frequency and severity. Furthermore, inconsistencies in treatment between immunocompetent and immunosuppressed patients complicate the issue. Many small therapeutic studies conflict with meta-analyses over the effectiveness of treatment in immunocompromised patients. While our chart review presents promising data, additional randomized control experiments are required to fully understand the efficacy of parasitic treatments, especially with regards to combination therapy.
